# Supplementary figures and images for: scAnt—an open-source platform for the creation of 3D models of arthropods (and other small objects)
Source: PeerJ. 2021 Apr 12;9:e11155. doi: 10.7717/peerj.11155 (PMC8048404; doi:10.7717/peerj.11155)

A

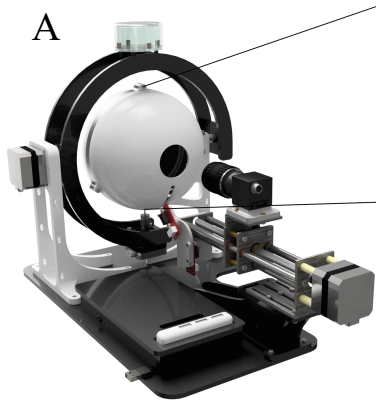

B

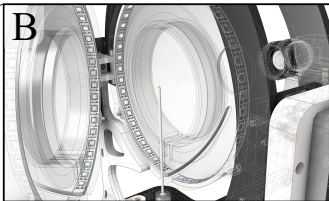

C

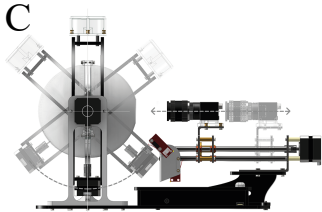

D

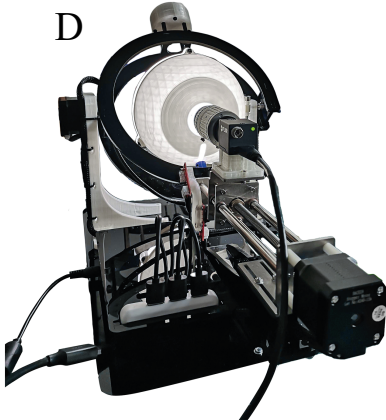

Supplement: Supplemental Information 1 — (A) Assembled scanner, excluding wiring. (B) Inside of the illumination dome, with the insect pin (vertical rotation axis) at its centre, and a circular array of LEDs in both halves of the spherical illumination dome. (C) Range of positions for the horizontal axis and camera, as controllable via the stepper-controlled camera slider provided in the graphical user interface of scAnt (see Fig. 2). (D) Photograph of the scanner as built. [file peerj-09-11155-s001.pdf]

A

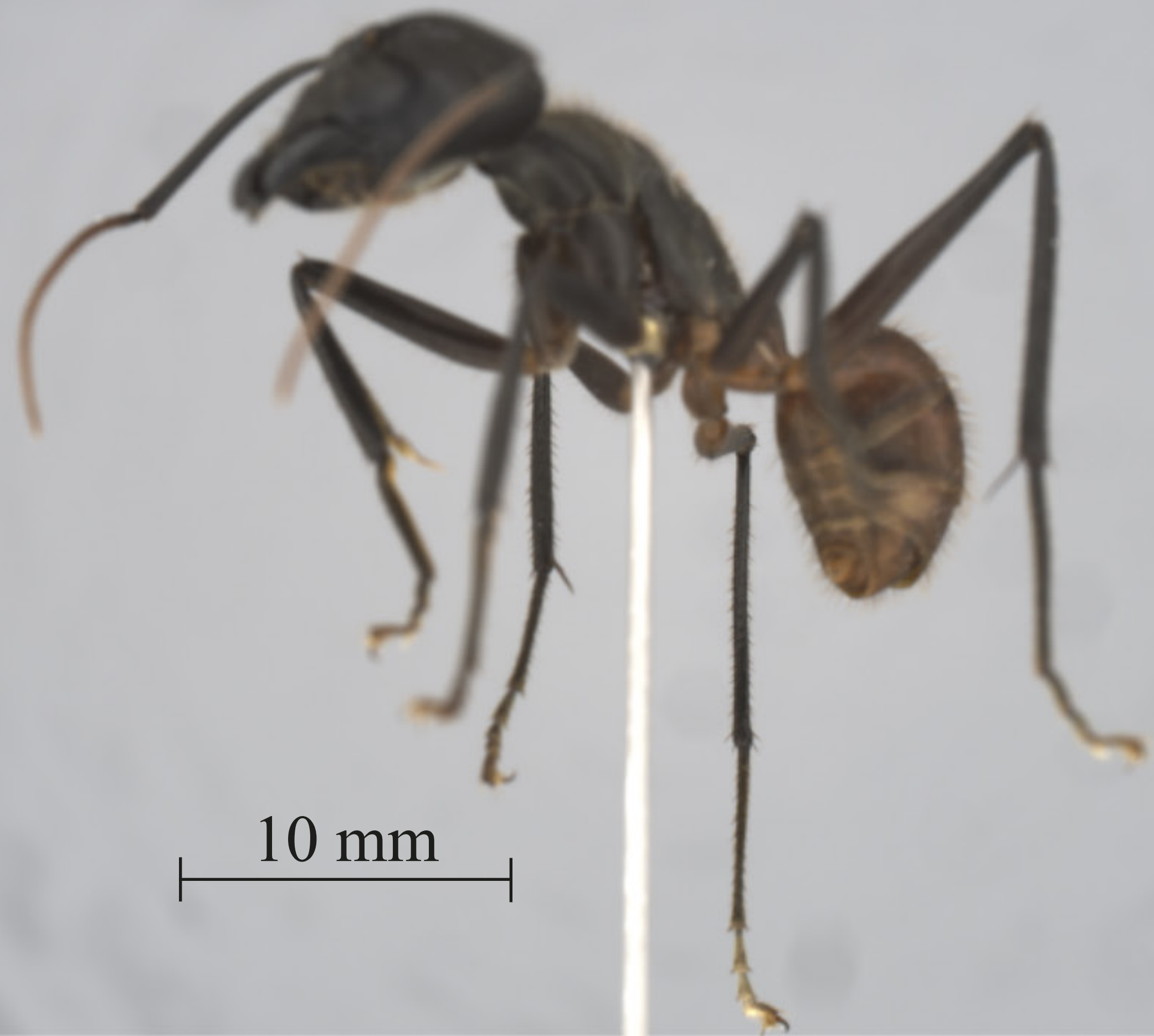

B

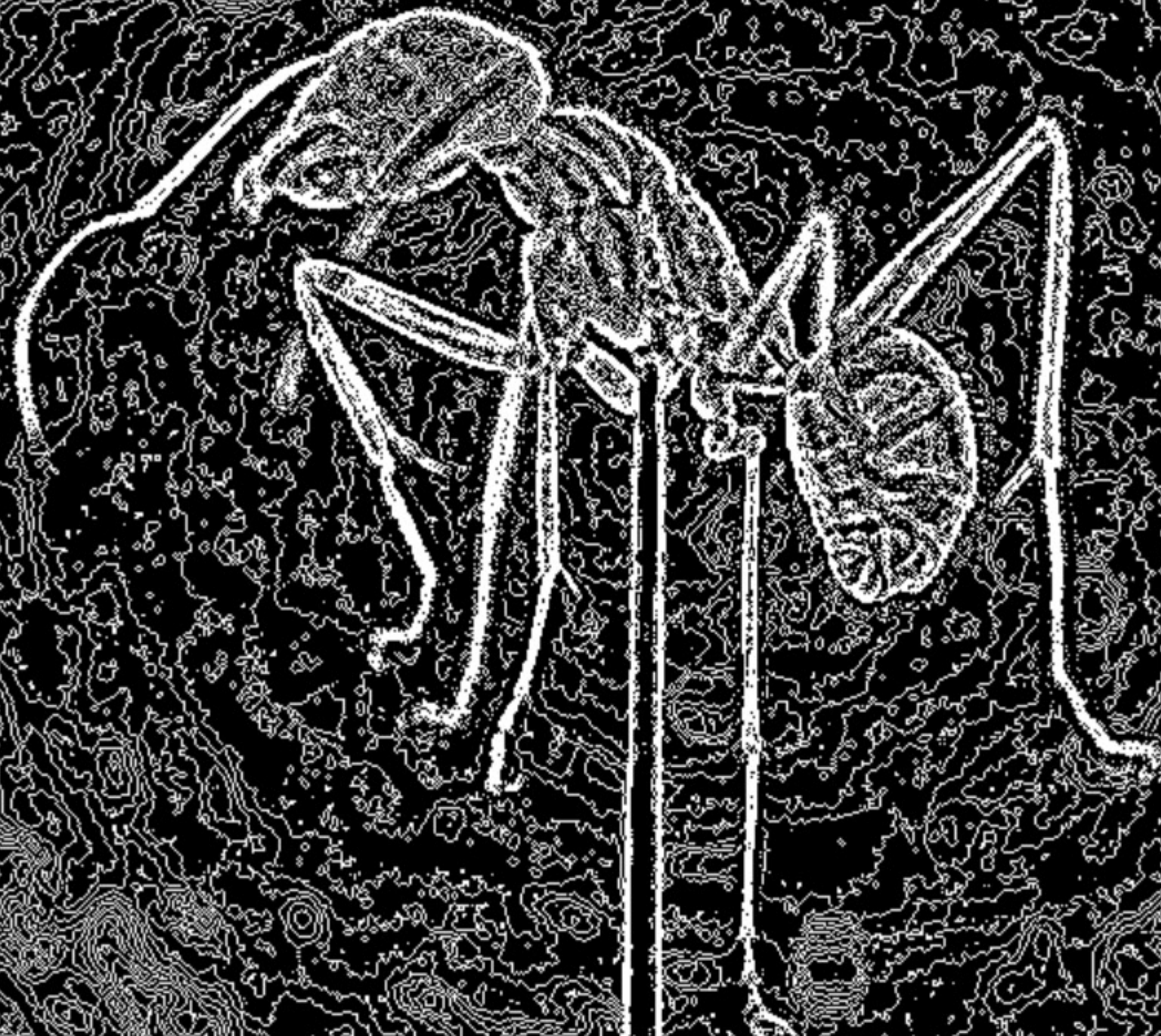

Supplement: Supplemental Information 2 — Prior to image stacking, images which do not include relevant in-focus pixels are discarded in an automated process. (A) Image of a Dinomyrmex gigas specimen; the focal plane is approximately aligned with the hind legs. (B) A Laplacian filter can be used as a simple edge detector, as it highlights areas with a high local variation in intensity. Sharp areas result in greater variance so that the variance of the Laplacian can be used as a suitable scalar proxy for the fraction of the image which is in focus. [file peerj-09-11155-s002.pdf]

A

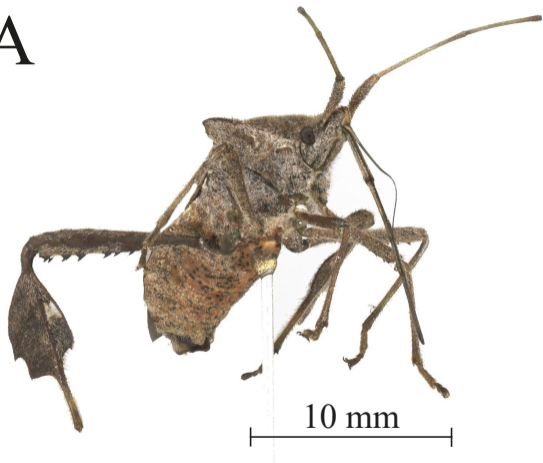

B

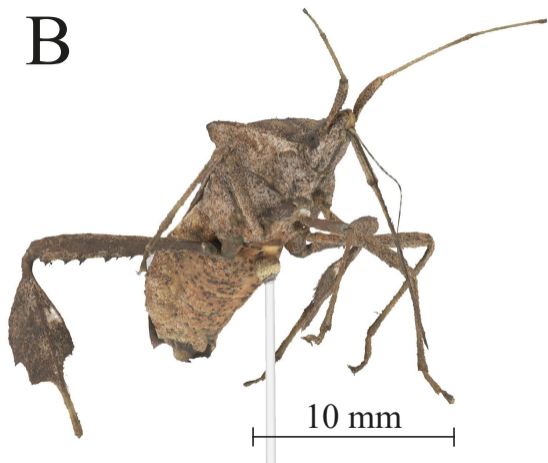

Supplement: Supplemental Information 3 — The lighting of the scanner, as well as the camera parameters were replicated in blender v2.8 and rendered with Cycles to qualitatively inspect the models for evidence of distortion as a result of the reconstruction process. [file peerj-09-11155-s003.pdf]
