# Supplementary material for: scAnt—an open-source platform for the creation of 3D models of arthropods (and other small objects)
Source: PeerJ. 2021 Apr 12;9:e11155. doi: 10.7717/peerj.11155 (PMC8048404; doi:10.7717/peerj.11155)
Supplement: Supplemental Information 4 — (A) EDOF image captured with regular, flat lighting. (B) EDOF image captured with backlighting only. (C) Mask generated from the flat lighting EDOF image via random forest-based outline detection. (D) Hand-annotated ground truth mask generated from the flat lighting EDOF image (A). (E) Mask generated from the backlighting EDOF via backlight thresholding as described by Ströbel et al. (2018). [file peerj-09-11155-s004.pdf]

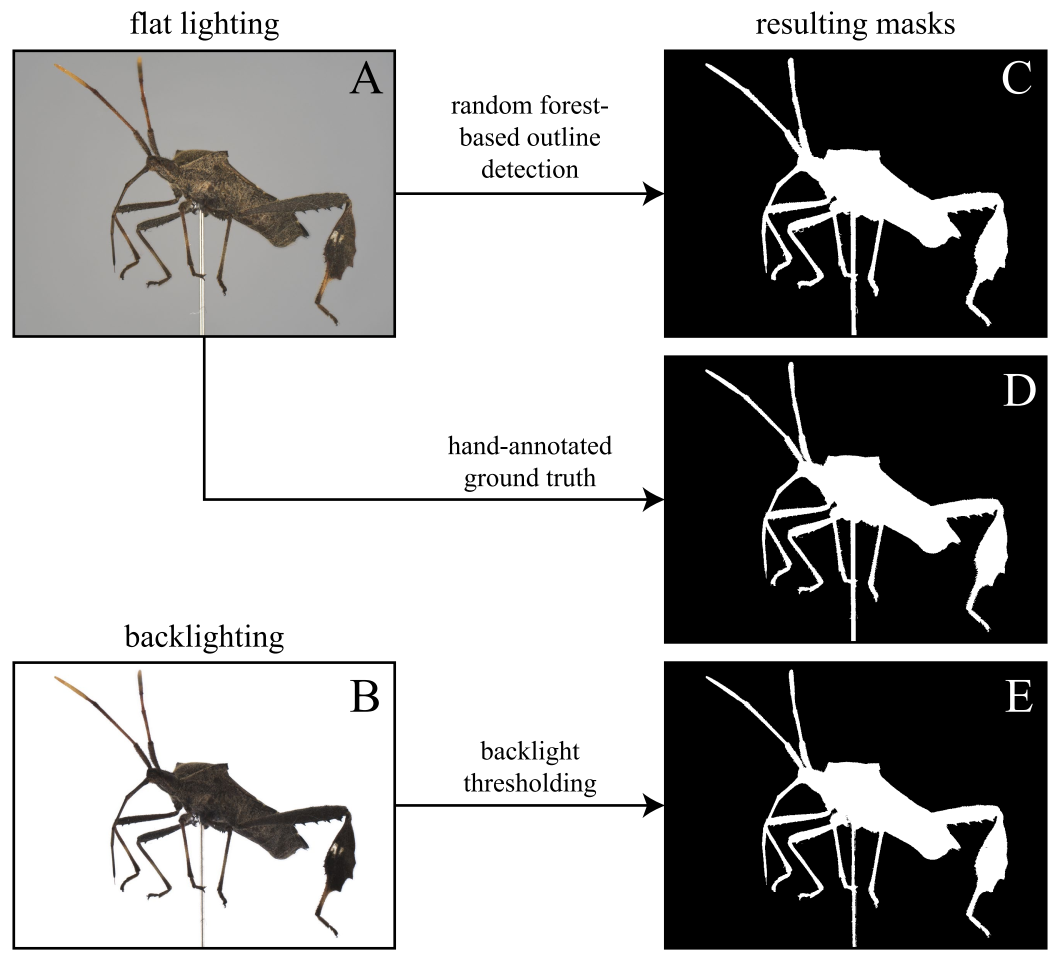
flat lighting
resulting masks
A
C
random forest-based outline detection
D
hand-annotated ground truth
backlighting
B
E
backlight thresholding
