# Supplementary material for: scAnt—an open-source platform for the creation of 3D models of arthropods (and other small objects)
Source: PeerJ. 2021 Apr 12;9:e11155. doi: 10.7717/peerj.11155 (PMC8048404; doi:10.7717/peerj.11155)
Supplement: Supplemental Information 5 — 31 uncompressed images for the flat lighting and backlighting EDOF images were captured at 20 MP resolution. The masking accuracy was computed by comparing the generated mask of each method to a hand labelled ground truth mask (see Fig. S4). The processing times refer to the combined time of the stacking and masking. The total file sizes refer to the combined amount of hard-drive space required to store all original and processed images. [file peerj-09-11155-s005.docx]

| method | masking accuracy | Capturing time [s] | Processing time [s] | Total file size [MB] |
| --- | --- | --- | --- | --- |
|  |  |  |  |  |
| backlight masking | 0.9902 | 95 | 564 | 1035 |
| random forest + adaptive thresholding | **0.9922** | **47** | **291** | **802** |
